# A New Identity: Enhancing Life Skills and Work-Readiness for Those With a Sexual Offending History

**DOI:** 10.5964/sotrap.14531

**Published:** 2024-10-15

**Authors:** Rachel Ogden, Emma Tarpey

**Affiliations:** 1 Psychology Department, Manchester Metropolitan University, Manchester, United Kingdom; University Medical Center Mainz, Mainz, Germany

**Keywords:** sex offenders, Good Lives Model, desistance, life skills, community, interpretive phenomenological analysis

## Abstract

The purpose of this research was to examine the experiences of individuals, with a sexual offending history, who have engaged with a community-based life skills and work-readiness programme. With a view to understand the impact of such programmes on their rehabilitation and to inform a growing body of research around the approaches needed for promoting rehabilitation within this group. Semi-structured interviews were conducted with four participants about their experiences of accessing a community-based charity and three themes were identified using Interpretive Phenomenological Analysis: “a place to belong without fear or judgement;” “creating an identity away from offending;” and “space and time to realise own potential.” These findings were discussed in relation to the Good Lives Model; previously identified protective factors for people with a history of sexual offending; and desistance research. The research offers a number of recommendations for practice which can be used by organisations to help them develop an inclusive and meaningful approach to supporting individuals with a history of sexual offending.

## Abstract (plain language summary)

**Background:**

There is a growing body of research into rehabilitative approaches for men with a sexual offending history, with it being widely accepted that strengths-based approaches are more successful than punitive. Life skills and work-readiness programmes are offered as a rehabilitative approach within the criminal justice system, however previous research has either not distinguished between offence types or have excluded individuals with a sexual offending history altogether. Therefore, there is limited research on how life skills and work-readiness programmes are experienced by individuals with a history of sexual offending. This research aimed to explore the experiences of individuals with a sexual offending history who had engaged with a life skills and work-readiness programme as part of a community-based charity.

**Why was this research done?:**

The highest level of sexual offences was recorded in the UK in the year ending March 2022 (195,315) and the number of sexual offences recorded by police has been increasing since 2012 (53,519). This means that there is an ever-increasing number of people in the community who have a history of sexual offending. It is important for us to understand how we are able to support them to live an offence-free life. This is crucial in preventing future harm from offending and preventing future victims of sexual offences.

**What did the researchers do and find?:**

The researchers interviewed men with a sexual offending history about their experiences of attending a community-based charity programme that delivers life skills and work-readiness courses. The charity supports a mixed community whereby not all the people who attend have an offending background or a history of sexual offending. The researchers found that the participants reported positive experiences of attending these programmes. They reported that having a place to belong where they were not judged enabled them to develop constructive support networks and a sense of community. This allowed them to create a new identity away from their offending past. The programme gave them the space and time to realise their own potential and achieve their goals. This was achieved by having a positive structure and routine; being able to develop healthy techniques to manage their wellbeing; and setting and achieving realistic goals.

**What do these findings mean?:**

The findings of this study identified that engaging in the community programme supported the participants’ rehabilitation. Organisations and services looking to support change for those convicted of sexual offences should consider the following recommendations to facilitate and encourage engagement: 1) Creating a safe place for people to attend by considering: the physical environment; the values and ethos of the service; prioritising health and wellbeing; and having robust and informed risk management processes. 2) Focus on identity change by offering person-centred, non-judgemental support that provides social inclusion through a needs-led approach. 3) Offering meaningful opportunities with personalised goal-focussed support and an opportunity to learn new skills with no time constraints.

## Highlights

- Participants report positive experiences of accessing community-based programmes.
- The community programme appears to support the move towards strength-based approaches to contribute to desistance from further offending.
- The community programme environment encourages the development of pro-social identities.
- The research supports a strengths-focussed approach for this cohort.

Desistance is a process by which people with offending backgrounds work towards leading a non-offending lifestyle and includes stopping and refraining from offending (Bersani & Doherty, 2018). Life skills and work-readiness programmes have been found to have a positive impact on individuals with an offending background with several studies demonstrating their effectiveness (Clark & Duwe, 2015; McKenzie & Tarpey, 2020). Life skills programmes offer strengths-based approaches that aim to build personal management skills to help people deal effectively with the challenges of life (Clark & Duwe, 2015) and work-readiness programmes have been developed to provide people with skills and confidence that would enhance their chances of gaining employment (Ford et al., 2015). McKenzie and Tarpey (2020) studied the experiences of people with any offending history who accessed life skills and work-readiness programmes. They found these programmes facilitated the desistance process with participants reporting a “need to change;” “changing identity;” “giving back to the community;” and “a sense of belonging” as outcomes to attending the programmes.

Jolley (2018) suggested that without addressing basic life skills, rehabilitation for people with an offending background could be severely limited as these life skills help individuals to develop the necessary tools to assist them in everyday life and are fundamental to their rehabilitation. The environment in which these programmes are delivered is important to consider, Psychologically Informed and Planned Environments (PIPEs) are widely used within the criminal justice system and aim to create a safe and supportive environment to facilitate rehabilitation (Benefield et al., 2017).

Those with an offending history can be faced with barriers when trying to secure employment (Zakaria et al., 2018) and developing employability skills has been identified as a key part for successful desistance in creating a non-offending identity (Cherney & Fitzgerald, 2016; Rhoden et al., 2022; Visher et al., 2011). However, Newton et al. (2018) identified that programmes which incorporate a broader approach to life skills, rather than just focussing purely on employability skills, are more likely to have a longer-term impact on a person’s ability to lead a non-offending life.

Whilst there has been extensive research into desistance from general offending, there is comparatively less research exploring desistance from sexual offending (Harris, 2021). Individuals with a sexual offending history have been overlooked in previous studies of desistance, either because the researchers fail to distinguish between offence types, or because they exclude individuals with a sexual offending history from the research altogether (Harris, 2017).

Research in this area has increased over the years (Lussier et al., 2023). There is a growing body of research that demonstrates that most men with a history of sexual offending eventually stop their offending behaviour (Cooley, 2022; McCartan & Richards, 2021; Richards, 2021), which is supported by the reoffending rates for individuals who have committed sexual offences being the lowest of any type of offending at 12.4%, compared to 24% for general offending (Ministry of Justice and National Statistics, 2023). However, it is common for individuals who have committed sexual offences to be treated differently to individuals with a non-sexual offending history within the criminal justice system (Harris, 2021). The current approaches to community risk management often include extra notification requirements, restrictions, polygraph testing, and limitations on housing and employment options (McCartan & Richards, 2021). These policies can often contribute to an increase in risk as they reduce a person’s opportunity to change; identify them as ‘other’; and limit their ability to be included in society (McCartan et al., 2021); as they are at odds with the strengths-focussed, pro-social principles of rehabilitation. Therefore, a shift from a punitive to a rehabilitative approach for individuals with a history of sexual offending is needed (McCartan, 2021).

A widely accepted model of rehabilitation is the Good Lives Modes (GLM) (Ward & Marshall, 2004). The GLM presents a holistic and strengths-focussed approach to rehabilitating individuals who have committed sexual offences. It focusses on the importance of developing primary human goods such as excellence in play; work and agency; community; and creativity. The GLM suggests that any intervention should be viewed as an activity that adds to an individual's personal functioning. This aims to include them as a functioning member of society rather than restricting a person’s activity by identifying deficits that require management or treatment. This is supported by Farmer et al. (2015) who identified that one major factor in desistance from sexual offending was having optimistic plans for the future, suggesting a strengths-based focus on rehabilitation of individuals with sexual offences is needed.

De Vries Robbé et al. (2015) agreed that a strengths-based approach such as the GLM is key in understanding the rehabilitation process for individuals convicted of sexual offences. They further suggested several protective factors that support desistance from sexual offending. These include: a constructive social and professional support network; engagement in employment or constructive leisure activities; and goal-directed living. It was concluded that risk assessments of those who are at risk of sexual offending are predominantly deficit-focussed, and risk management could be more effective if the focus was strengths-based and included protective factors.

It has been suggested that those who have been convicted of sexual offences share more similarities than differences with individuals with general, nonsexual offences (Harris, 2021; Harris et al*.,* 2009). Whilst it can be agreed that the strengths-based approach to rehabilitation supports desistance for individuals with a history of sexual offences as well as those with general offences, it is important to recognise and understand the challenges and obstacles individuals convicted of sexual offences face (Harris, 2017). These experiences are often more challenging to overcome due to the stigma and restrictions they face (Brown et al., 2007).

Research exploring the resettlement of those with a history of sexual offending who had served time in custody highlighted the various barriers they may face when re-settling into the community (Mills, 2012). This research identified a lack of formal support or programmes to address problems such as: isolation; lack of sense of community; and feeling unemployable. These challenges and problems are in contrast to the protective factors and primary needs highlighted as being important to an individual’s desistance from sexual offending (de Vries Robbé et al., 2015; Farmer et al., 2015; Ward & Marshall, 2004).

McCartan (2021) suggests that the integration of people who have committed sexual offences into the community should focus on the development of the person rather than focussing on the offence and emphasises that sexual offending should be seen as a health, life course and wellbeing issue. Identifying practical approaches to rehabilitation that look to address these issues in strength-focussed ways could provide opportunities for individuals with a sexual offending history to work towards desistance. Therefore, suggesting that a combination of life skills and work-readiness programmes delivered in a safe and supportive environment could offer a comprehensive strengths-based approach to supporting people with a sexual offending history.

The present study explores the experiences of individuals with a sexual offending history who have engaged in a community-based life skills and work-readiness programme.

## The Present Study

The aim of this research was to explore the experiences of individuals with a sexual offending history who had engaged with a life skills and work-readiness programme as part of a community-based charity. As this research was interested in exploring the experiences of individuals engaging with a life skills and work-readiness programme, a qualitative research design was deemed most appropriate. Interpretive Phenomenological Analysis (IPA) was the analytical method used. IPA is an approach that examines how people make sense of major life experiences and is focussed on analysing experiences (Smith et al., 2022).

## Method

## Setting

All participants were members of a community-based charity that supports a mixed community whereby not all the people who attend have an offending background or a history of sexual offending. A person attending may have experienced challenges with their mental health, substance misuse, homelessness, or offending background. The charity offers life skills and work-readiness programmes and focusses on building people’s strengths, it aims to support adults to make meaningful and realistic changes to their lives.

Members of the charity are empowered through courses, activities and work experience that builds confidence and skills at their own pace, with their being no time limit to how long they can attend. Through this process members can access the support and guidance to make their own choices about their future and goals.

The charity provides a safe, friendly community where people feel they belong and can make new connections. Trauma-informed practices underpin all decisions and there is no hierarchical structure to the charity where the ethos sees staff, volunteers, and members as equal within the community who contribute in different ways. The staff and volunteer team incorporates people who have previously accessed the charity for support or who have lived experiences in their background.

The environment that is created is underpinned with robust and informed risk management. The charity refers to this as “safety-planning” as it focusses on what measures can be put in place to create a safe environment for a person to attend, rather than what restrictions can be put in place to manage the risk of a person attending. These safety-planning meetings occur weekly and involve staff of different levels, including senior management, to make a collective and agreed upon decision about whether the charity can provide a place for a person. In circumstances where it is decided a person is not able to attend, the charity will provide a plan to that individual of what they would need to see in place in order for the person to be able to safely attend, and will give a timeframe for when this will be reconsidered.

This process is especially important for individuals attending with a history of sexual offending to ensure that the safety of the person and the safety of other members is prioritised. This process offers a holistic approach that looks at a person’s current circumstances and does not focus solely on the person’s offence.

## Participants and Recruitment

All participants were engaged with the host charity and were selected using purposive homogeneous sampling due to the participants needing to share certain characteristics. The inclusion criteria involved participants who were adult male with a previous conviction for a sexual offence. Previous research has focussed on males with a sexual offending history (Cooley, 2022; McCartan & Richards, 2021; Richards, 2021) and there has been a reported need for a gender-specific approach when working with people with sexual offending histories (Williams et al*.,* 2019). Participants were also required to be no longer on licence as they will have been engaging with the charity for a longer period of time and be better able to reflect on their experiences. The host charity nominated a gatekeeper who identified and contacted potential participants. Five participants were initially recruited for the research, with four successfully completing the research interviews. A small sample size was used because IPA is an idiographic approach which aims to analyse and understand a particular phenomenon in specific contexts (Smith et al., 2022). The gatekeeper’s role within the organisation was that of managing risk and safeguarding of programme participants therefore participants did not need to disclose their history to the researcher to take part. All participants were male, aged between 30 and 74 years old, and had been attending the charity for between 3 and 7 years. Two participants had previously spent time in custody and all participants referenced having experienced mental health challenges at some point post-conviction.

## Design and Procedure

This research used a qualitative research design using semi-structured interviews as a method of collecting data which allowed for flexibility in the interview process (Willig, 2001). A critical realist approach was adopted as the research was capturing participants’ lived experiences (Maxwell, 2012). An interview schedule was created using the guidelines of IPA analysis by Smith et al. (2022) with each interview taking approximately 30 minutes. The interview focussed on three main areas: experiences prior to attending the programme; experiences of attending the programme; and thoughts and feelings about the future. The design of these questions considered previous research on the topic (Maruna, 2001; Ward & Marshall, 2004). The interviews took place at the charity’s premises and were recorded using a voice-recorder and subsequently transcribed by the interviewer. The interviewer for this current study was also a practitioner at the programme at the time of the research. This role involved supporting the participants during their time at the programme and therefore they had a prior relationship with the participants. One of the most important aspects of conducting an IPA interview is for the interviewer to establish a rapport with the participant, where the participant feels comfortable and can trust the interviewer (Smith et al., 2022). Because there was a prior relationship between the interviewer and the participant, this meant that there was an established rapport and level of trust. This could allow for a meaningful conversation to take place, especially when discussing a sensitive topic for the participants.

## Ethics

Manchester Metropolitan University granted ethical approval, and permission was granted by the Chief Executive of the charity for the research to be carried out with their programme’s participants on the charity’s premises. Participants gave their informed consent to take part and were made aware of their right to withdraw at any time. Participant data was pseudonymised during transcription and no personal identifiable information was connected to the research data. Due to the inclusion criteria, the researcher did have an awareness of the nature of the participants offending background, however they were not provided with specific details and no participant was required to make a disclosure in order to take part in the research. Participants were made aware that the charity would not be named in any future publication of the findings to further protect their identity.

## Analytical Method

The analytical strategy of IPA outlined by Smith et al. (2022) was used to generate the outcomes of this research. IPA was the chosen analytical method as it provides a detailed examination of someone’s experience. Participants are selected because they can provide a perspective of the area being studied and IPA provides an analysis on this specific perspective rather than an analysis of an entire population. A detailed case-by-case analysis was undertaken by the researchers which aimed to allow the researchers to write in detail about the area of study.

This process involved the primary researcher reading and re-reading the data to become familiar with the narratives. The researcher then focussed on making exploratory notes aiming for a comprehensive and detailed set of comments. The next stage involved consolidating these initial thoughts by creating experiential statements. The experiential statements related directly to each participants’ experiences. The researcher then began searching for connections across these experiential statements to create Personal Experiential Themes (PETs). The PETs are personal themes for each participant. These PETs were classified as the subordinate themes in this research and finally, patterns and themes across these PETs were identified to develop the Group Experiential Themes (GETs) which were classified as the superordinate themes for this research.

## Findings and Discussion

Through conducting IPA, three superordinate themes were identified. These were: a place to belong without fear of judgement; creating an identity away from offending; and space and time to realise own potential.

### A Place to Belong Without Fear or Judgement

This theme captures the value participants placed on the positive environment of the programme and how this supported their engagement and progression. All participants placed an importance on feeling safe and supported, not judged, and belonging to a community as the main reasons they engaged with the programme.

#### A Person-Centred Environment

This subordinate theme highlights the importance participants placed on the nature of the environment of the programme. A person-centred environment is one where the focus is on the needs of the individual (Murphy et al., 2013) and it has been suggested there is little success in adopting a ‘one size fits all’ approach when working with individuals who have committed sexual offences (Laws & Ward, 2011).

The participants’ narratives placed an importance of feeling safe and not judged within the environment and how this impacted on their motivation to remain offence-free:

*“The main thing is the environment, it cheers me up when I am here, especially on my worse days…being here has literally reduced my risk of reoffending by a lot as I’m not at home stewing over my own thoughts.”*

*(Gary: Line 78–81)*

*“Being part of the criminal justice system can make you feel less than human, particularly with sex offences…that label will always be there. One of the things that being here has done for me is it made me feel human again.”*

*(William: Line 78–87)*

William highlights the importance to him of not being judged for his offending background. James and Mike express how the fear of being judged can be a cause of worry when accessing new environments:

*“It’s the first thing that goes through my head when I go somewhere new, am I going to get judged here?”*

*(James: Lines 52–53)*

*“I think because of the offence it kind of destroyed the life that I had, I was in fear of people recognising me and knowing who I was.”*

*(Mike: Lines 22–23)*

Accessing an environment where they are not being judged is strongly felt throughout the narratives of the participants and this is an important aspect of participants feeling safe when attending the programme:

*“It makes me feel included and accepted.”*

*(William: Line 175)*

*“That’s the thing about coming here, not feeling like I’m being judged…I feel normal coming here.”*

*(Mike: Lines 43, 47)*

*“Once you get through the doors you don’t have to worry about being judged, you’re perfectly fine.”*

*(James: Lines 54–55)*.

Blagden et al. (2016) interviewed prisoners with sexual offences and identified how prisoners placed an importance on their environment being non-judgmental and safe. This environment was found to have contributed to positive change in prisoners. Furthermore, Westaway et al. (2017) suggested that individuals who are stigmatised due to their backgrounds are likely to internalise these negative views suggesting that a person-centred approach is an important aspect of creating a safe environment. These findings are reflected in the current research as the participants’ narratives demonstrate the importance placed on how the person-centred environment gave them a feeling of safety which helped them to attend and engage initially within the programme.

#### Developing Meaningful and Constructive Support Networks That Promote a Sense of Belonging

This subordinate theme highlights the importance placed on being able to access and develop support networks by the participants. Participants reflected on support they were receiving prior to attending the programme; with the narratives showing that support was mainly from statutory agencies such as the probation service, the job centre, or housing key workers. James reflected on how this was not having a positive impact, suggesting the support being offered wasn’t considering his background and didn’t offer the specialist non-judgemental support that James required:

*“My weeks were just mainly probation and the job centre; it was just them. And I felt really low, I was just doing them things and it made me feel like I didn’t fit, like I didn’t belong anywhere.”*

*(James: Lines 33–25)*

In comparison, participants talked positively about the support they were able to access from the programme, reflecting on there being no barriers and a feeling of not being judged. This support was constructive in helping participants to improve their motivation and engagement.

*“There’s no closed doors…I can talk to anyone I want to. If I need to talk to any of the staff, I can just ask. There’re no barriers to go through.”*

*(Mike: Lines 70–72)*

*“They’ve helped me out so much. Not just keeping me offence free but they’ve helped me getting experience in admin as well as all the help that I’ve had to get to university.”*

*(Gary: Lines 117–119)*

The narratives also indicate the impact on participants of developing positive social networks with peers. There is little discussion about any social networks or support from friends prior to attending the community programme. Mike described how isolation was a challenge and a factor in being motivated to change.

*“I was going through some troubles with isolation…I was very very isolated, very very lonely.”*

*(Mike: Lines 18, 27)*

The participants all discuss how accessing the community programme has combatted prior feelings of isolation through being able to meet people, develop friendships, and give them a sense of purpose. Gary explains what he enjoys most about attending the programme:

*“It’s meeting new people, out of all of the time I’ve been coming here no one comes in and is like ‘I don’t like that person,’ they enjoy seeing me and I enjoy seeing them. It gives me purpose in life.”*

*(Gary: Lines 71–74)*

For some participants, they describe how their lives outside of the programme can still be isolating so they place a real importance on belonging to a community where they can be around others and not be judged. Both William and Mike explain how important attending the programme is to their lives:

*“I feel normal coming here, but I still suffer with isolation because I live in a shared house and I don’t see anyone...I don’t always come here because I get stuff out of it, sometimes I come because it’s physical human contact, someone to talk to, someone to chat to, someone to have a laugh with or just to listen.”*

*(Mike: Lines 47–53)*

*“One of the things that is nice about coming here is being part of a group, in which you are accepted. I wouldn’t have that unless I came here…my circle of acquaintances is very small, so it’s nice to come here and have that interaction and have that in my life.”*

*(William: Lines 165–167; 171–172)*

The participants’ narratives describe the value they each place on having meaningful contact with others, whether that is socially or professionally, thus creating a sense of belonging. Connecting to a wider social group is one of the primary human goods in the GLM (Ward & Marshall, 2004) and developing a constructive social and professional support network is a protective factor in desistance from sexual offending (de Vries Robbé et al., 2015). Furthermore, life skills programmes offer individuals an opportunity to develop a sense of belonging (McKenzie & Tarpey, 2020). Malhotra and Gussak (2021) found an important aspect of strengths-based interventions for individuals with a sexual offending history was the sense of belonging that it created. The importance of these networks and the impact on creating a sense of belonging was further explored in the subordinate theme of “a sense of community and relatedness to peers.”

#### Sense of Community and Relatedness to Peers

This subordinate theme highlights the participants’ feelings around being part of a community and being able to relate to others within that community. The GLM describes relatedness and community as being important aspects of desistance from sexual offending (Ward & Marshall, 2004). Participants were asked how developing positive social networks had impacted them and their engagement. James explains what initially encouraged him to engage in the programme:

*“It was somewhere to be where I could be part of a group with people at the same level, with people who have gone through the things that I’ve gone through, well not necessarily exactly what I’ve experienced, but people who also had mental health problems and things like that. We were all at the same level and they were all very welcoming.”*

*(James: Lines 66–70)*

Being able to relate to others in ways not solely focussed on his offence was also important for Mike. Being able to move away from the identity of his offence and recognise that others, who may not have offended, faced similar challenges to himself, allowed him to feel that he was able to be himself:

*“A lot of people are in the same boat as me with the isolation, the mental problems, people that might see themselves as different outside. But when they come here it’s a different face they put on, their face that they want to be not what they are on the outside.”*

*(Mike: Lines 102–105)*

James and Mike both describe how being able to relate to others is important to them and it plays a big part in their engagement with the programme by providing a safe environment for them to attend.

The narratives demonstrate the importance placed on feeling part of a community. Both Mike and William describe how one of the most important parts of the programme for them was the sense of belonging:

*“The feeling of belonging, I feel that I should be here, and I’m wanted here. I think that’s what the best thing is.”*

*(Mike: Lines 79–80)*

*“…you have unconditional positive regard towards individuals…they are still regarded as part of the team. It is that that has been the most important to me as an individual.”*

*(William: Lines 96–99)*

Sexual offending is often seen as ‘different’ to other types of offending, with there being a hostility towards individuals who have committed sexual offences (Thomas, 2015). This exclusion from society can cause these individuals to feel a lack of sense of community (Mills, 2012). Cloud and Granfield (2009) suggested that exclusion on an interpersonal and societal basis can be a barrier to an individual’s recovery. The current research demonstrates how feeling a part of a community and being able to relate to others was important to the participants’ journey. Best et al. (2018) demonstrates that belonging to a community encourages and supports positive change towards an individual’s desistance.

### Creating an Identity Away From Offending

This theme demonstrates how the participants discussed their identity and the importance they placed on moving forward from their offending backgrounds. Being able to develop a pro-social identity has been found to be important for an individual’s journey towards desistance (Maruna, 2001). The narratives show by having a motivation and recognition of a need to change, the participants were able to benefit from accessing opportunities to learn; develop; and progress, in order to create an identity and life away from offending.

#### A Motivation and Recognition of a Need to Change

This subordinate theme highlights how participants recognised a need to change prior to attending the programme. The narratives show how participants wanted to make a change but were unsure on how to do this. Gary explains how he was unsure at the beginning what he would experience by attending the programme, but he talks about a desire to keep his risk low and not reoffend:

*“I didn’t really have a clue on what I would get out of coming here…it was just somewhere to spend time, meet new people and keep me lower risk really.”*

*(Gary: Lines: 53–57)*

Mike discusses how having experienced isolation prior to attending the programme meant that he felt a need to make a change to his life. When asked what gave him the courage to attend for the first time he explained:

*“I don’t really know why, I just thought I’ve got to do it, I’m here, I’ve got to do it, I’ve got to try. Or I’d just go back to the shelter and sit there and mope. So, I came in.”*

*(Mike: Lines 33–34)*

On reflection, the participants describe how they didn’t know what they needed to do but they recognised they wanted to change and move away from their offending background. This suggests the participants were looking to develop the life skills needed to help them meet their goals. James describes this feeling and what his motivations were when he first attended:

*“I’d say a desire to change myself, get back to the way people saw me before everything bad happened…because I had gone right back into my shell, so to speak, I was very shy, I had no confidence.”*

*(James: Lines 27–30)*

The narratives demonstrate an internal motivation to make a positive change to their lives prior to attending the programme. Having a motivation to change is a key step in an individual’s journey towards desistance (Farrall, 2022). Bushway and Paternoster (2014) argue that identity and cognitive changes within the offender must precede external and structured support in order to effect change. This supports the current research whereby the narratives suggest that these cognitive changes had occurred prior to participants engaging with the programme.

#### Opportunities to Learn and Develop New Skills

This subordinate theme builds on the participants’ motivation to change as the narratives highlight how the participants wanted to change but were unsure on how to do this. Aresti et al. (2010) demonstrated that self-change was a positive experience for people with convictions allowing them to move on from their negative past experiences. The narratives show, how prior to attending the programme, participants felt they were not equipped with the tools they needed to progress and develop. Gary describes the struggles he had with applying for work:

*“The thing I found difficult was probably just applying for jobs, it was all new to me, you know, disclosing and that. I didn’t have a clue on what to do or how to do it.”*

*(Gary: Lines 36–38)*

The narratives describe a variety of different opportunities that the participants were able to access at the programme. Participants place an importance on how accessing these opportunities have given them the chance to develop skills.

*“There’s no closed doors, I can have a go at anything I want to… It makes me feel good because I get to talk to people, I get to do things.”*

*(Mike: Lines 70, 76)*

*“When I saw the stuff that I could do I thought this is a chance to broaden my skills and improve on certain areas.”*

*(James: Lines 44–46)*

*“When I did the admin course it gave me the basic skills of something I could put to work, it was good...the best thing was I could get experience through volunteering after the course.”*

*(Gary: Lines 61–68)*

Participants were asked where they thought they would be now if they had never accessed the programme. Their answers highlight feelings of low mood and how being provided with the opportunities to learn and develop has had a significant impact on the participants lives and their own identity.

*“If I hazard a good guess, I would probably be back inside serving another term for something else. Not for the same crime I committed before, but for something to do with other offences.”*

*(Gary: Lines 223–225)*

*“I don’t want to think about where I’d be now. I wasn’t in a good place before I came here, it would have been a downward spiral, I think.”*

*(Mike: Lines 139–140)*

*“I would have stayed where I was, where I was low. I wouldn’t have had the opportunities…I’ve had so many opportunities through coming to this place over the past three years. And just being able to do them is a blessing for me, developed my abilities that I didn’t know I had inside.”*

*(James: Lines 217–219)*

The GLM suggests that offences can occur as a result of individuals seeking inappropriate ways to obtain primary goods (Ward & Marshall, 2004). In the current research, participants were able to find appropriate ways of obtaining their primary goods by accessing constructive leisure programmes which have been highlighted as a protective factor (de Vries Robbé et al., 2015). Furthermore, Ward and Laws (2010) suggested that the process of self-change towards a pro-social identity is enhanced when individuals are provided with meaningful opportunities to learn and develop, as demonstrated in this current research.

### Space and Time to Realise Own Potential

This theme demonstrates how participants feel they were able to develop and realise their own potential through their experiences of accessing the programme. The narratives place an importance on the space and time needed for participants to achieve this. Having a positive structure and routine; being able to manage their own wellbeing; and set realistic goals, supported participants to realise their own potential.

#### Development of a Positive Structure and Routine

This subordinate theme highlights how participants placed value on developing a positive structure and routine having previously not known how to achieve this. The narratives suggest this being one of the main motivations for participants when they first attended the community programme.

*“I think at the time I wasn’t looking to achieve anything really, I just wanted to get back into a routine and things like that.”*

*(James: Lines 43–44)*

*“I didn’t really have a clue what I would get out of coming here apart from having somewhere to go and keeping me with a structure.”*

*(Gary: Lines 53–55)*

Gary further discusses how being a volunteer has given him structure and has enabled him to develop key work-readiness skills in managing his time and routine. He compares his time management skills prior to being a volunteer to his current routine:

*“There was a time before I came to the community programme where I would be up most of the night on my Xbox and stuff and I’d wake up late.”*

*(Gary: Lines 207–209)*

*“Coming here definitely helped me keep my days structured. Like before, I didn’t have an alarm set up but now to keep me in my routine I have an alarm set every day for 6am even if I’m not volunteering on that day.”*

*(Gary: Lines 216–218)*

Through analysis of the narratives, it was clear the importance placed on developing a structure and routine. By participating in well-structured programmes, individuals with sexual offences are more likely to be able to develop desistance strategies (Ward & Laws, 2010). This is conducive with research for people with non-sexual offences where it has been suggested that desistance is about developing a different set of routines that takes an individual away from places where they used to offend (Bottoms, 2014; Farrall et al., 2014).

#### Developing Healthy Techniques to Manage Own Wellbeing

This subordinate theme highlights the value the participants place on being able to better manage their own wellbeing and how this helps them to combat negative views of themselves. The narratives demonstrate how participants are more aware of their feelings about themselves and are now better prepared in being able to manage these. Gary and Mike describe how their self-belief has improved.

*“I still have issues with confidence, but I do have a better understanding of when I do put my mind to something I can accomplish it.”*

*(Gary: Lines 186–188)*

*“I don’t feel as bad about myself as I did when I first came out, there’s people I can talk to now even if it’s just to chat or I can actually come here and talk to someone in confidence.”*

*(Mike: Lines 85–86)*

Mike explains how having support and discussing his feelings with others has helped him to combat negative views he had about himself. Having negative self-worth is common among individuals who have a history of sexual offending (Farmer et al., 2015).

*“I was saying how I felt about myself and how I feel that I’ve got no self-worth, I’m not liked, no one likes me, and the tutor said, ‘you’re completely wrong on that, that’s not how I see you.’ … So other people might see me differently, it’s something I’ve got to work on myself, seeing me how other people see me.”*

*(Mike: Lines 94–99)*

James explains how by accessing the group sessions have helped with his wellbeing, even though he was unsure about their benefit before attending. He has been able to discover techniques to support his mental health:

*“It was the walking groups mainly and the wellbeing groups where you focussed on things like breathing…at the start I felt a bit uneasy but as the weeks progressed, I got more comfortable and confident.”*

*(James: Lines 59–63)*

*“I think the walking groups is where I’ve benefitted the most…it makes me feel a lot better…people had told me it was good for me, but it wasn’t until I went out on the small walks that I thought ‘they’re not wrong here’. It was working for me.”*

*(James: Lines 74–81)*

The GLM describes developing inner peace as one of its primary goods, whereby being free from emotional turmoil and stress can have a positive impact on desistance (Ward & Marshall, 2004). McKenzie and Tarpey (2020) identified that a similar programme for individuals with non-sexual offences helped participants to feel more confident and optimistic. The analysis in the current research shows how participants feel better able to manage their own wellbeing after attending the programme.

#### Setting and Achieving Realistic Goals in Their Own Time

This subordinate theme captures the progress that participants feel they have made whilst accessing the programme. The narratives all tell a story of how each participant has created a new identity through developing themselves and their skills by recognising their own potential, believing in themselves, and setting realistic goals to achieve this. This progress was evident in James’ narrative:

*“I would say that my goals have changed. Coming here has helped me get involved in other communities, other charities as well. One of the charities I’m involved in has given me experience in public speaking, which I never thought I would do and now I have the confidence to do it more…I think ‘past me’ would have said ‘you have got to be joking! You, speaking in public, get lost not a chance!’ That’s what I would have said, that’s what everyone would have said. I can’t believe I did it. But it went well, and I did it.”*

*(James: Lines 133–139; 166–168)*

The narratives demonstrate the value participants place on being able to set goals in their own time whilst feeling confident in being unsure about the future.

*“The future is still a bit grey, but there is always that bit of positivity by coming here and things like that. You learn about other places as well by coming here, you expand your horizons.”*

*(Mike: Lines 119–121)*

Being able to realistically plan for the future and maintain optimism is an important aspect of desisting from sexual offending (Farmer et al., 2015). Willis and Grace (2009) found better recidivism outcomes for prison leavers who had plans for the future than those who did not. McMurran (2002) suggested that in order to motivate people with an offending history to change their behaviour a focus on meaningful goals is important. The analysis of the current research emphasises how the participants were able to set goals at their own pace and how the process of achieving these goals allowed participants to gain confidence in their own potential.

Table 1 demonstrates the superordinate themes and their corresponding subordinate themes.

**Table 1:** Superordinate Themes and Their Corresponding Subordinate Themes

	Superordinate Theme		
	A place to belong without fear or judgement	Creating an identity away from offending	Space and time to realise own potential
**Subordinate Themes**	Person-centred environment	Opportunities to learn and develop new skills	Development of a positive structure and routine
	Developing meaningful and constructive support networks that promote a sense of belonging	A motivation and recognition of a need to change	Developing healthy techniques to manage own wellbeing
	Sense of community and relatedness to peers		Setting and achieving realistic goals in their own time

## Conclusion

This research highlights the positive experiences of participants, with a sexual offending history, accessing community-based life skills and work-readiness programmes. The findings demonstrate that by accessing an environment that allows participants to feel safe and supported, they have the space and time to create a pro-social identity and to develop key skills. This allows participants to feel able to develop the self-directedness to realise and achieve their own potential. These findings are supported by key desistance research that identifies the importance of safe and supportive environments (Blagden et al., 2016); belonging to a community (Best et al., 2018); developing a pro-social identity (Maruna, 2001); and having plans for the future (Farmer et al., 2015).

The current research supports the GLM’s strength-focussed approach to desistance whereby an intervention should be an activity that adds to an individual’s personal functioning (Ward & Marshall, 2004). Furthermore, demonstrating that individuals must develop in several key areas to experience positive outcomes (Ward & Laws, 2010). This includes the development of excellence in play; work and agency; community; and creativity.

Therefore, in conclusion, the programme offered a supportive environment that encouraged the development of pro-social identities in order to develop the primary goods of the GLM. The research demonstrates the positive experiences of accessing community-based life skills and work-readiness programmes for individuals with a sexual offending history, and this programme appears to support a move towards working with people convicted of sexual offences in a strength-focussed and trauma-informed way.

### Limitations

The findings of this study are overwhelmingly positive and suggest that those with a conviction of a sexual offence have a positive experience in attending life skills and work-readiness programmes. The lack of more negative themes could allow for speculation around the recruitment of participants; the small sample size; and the prior relationship between interviewer and participant.

The recruitment of the participants by the nominated gatekeeper could be considered a limitation of this research. Purposive sampling was used to identify participants, and this may have led to sampling bias as the nominated gatekeeper may have identified individuals who were highly engaged and more likely to speak positively about the programme.

The study only interviewed participants who had engaged in the programme, therefore the experiences of individuals who did not engage was not captured. It is recognised that the experiences of individuals who disengage from programmes is different to those who do engage (Bushway & Apel, 2012).

It could be argued that there are limitations to the generalisability of this study given the small participant numbers, however the purpose of IPA studies is to recruit small sample sizes so that the focus is on the quality of the experiences discussed rather than the quantity (Smith et al., 2022).

The primary researcher for this current study was also a practitioner at the programme at the time of the research. This role involved supporting the participants during their time at the programme. This was mainly advantageous to the research as it offered a unique insight into the community programme which allowed the researcher to identify the need for such research to take place. A key part of IPA analysis is for the interviewer and participants to have a strong rapport; the dual perspective as a practitioner and researcher meant that there was an existing rapport between the researcher and participants which allowed meaningful conversations to take place. However, it is important to note and be aware that their involvement in the programme may have increased the likelihood of subjective analysis. As participants had a prior relationship with the researcher, this may have also influenced their answers during the interview process.

It is important to consider the growing body of research that demonstrates that most men with a history of sexual offending eventually stop their offending behaviour (Cooley, 2022; McCartan & Richards, 2021; Richards, 2021) and consider the reasons as to why this might be. Cooley (2022) suggested that for individuals who truly desisted from sexual re-offending demonstrate cognitive transformations. This suggests an internal process of change rather than the impact of external support. However, Cooley (2022) also distinguished between “desisters” and “non re-offenders”, suggesting that “non-reoffenders” were able to manage their behaviour through strategies and external support. This supports the positive themes of this research as those who are “non-reoffenders” require the platform of support this community programme provides.

## Future Suggestions

A strong component of this research was exploring participants’ motivations for wanting to change prior to attending the programme. Exploring why some individuals decline or disengage from the programme may provide useful insights into motivation and the process of change within the type of service provision.

This research focusses on individuals who have a sexual offending history, however even within this label it relates to a variety of offence types. Future research could look more specifically at the experiences of individuals with specific types of sexual offences to aid further understanding of desistance.

### Recommendations for Future Practice

Services can consider the following recommendations if looking to develop similar themes when supporting people with a history of sexual offending. These recommendations have been suggested using the themes of this research and approaches the community programme currently adopts:

**Developing a Place to Belong Without Fear or Judgement:**
- The physical environment
  - The physical space needs to be welcoming, open, and trauma informed. Having ‘non-clinical spaces’ that offers an environment which differs from statutory services.
- Risk Management
  - Robust and informed risk management is key in creating an environment that is safe for everyone to attend.
  - Focussing risk management on safety planning rather than risk assessment means that the process is about what measures can be put in place to safely support someone rather than focussing on the restrictions.
  - The focus on safety planning allows for a holistic approach whereby decisions are made based on the person’s current circumstances and needs rather than the offence itself. This deeply embeds the principles of person-centred approach and ensures a service is creating a place that is safe for all.
  - Decision making and support whereby the person’s needs are seen as the priority and are not focussed on the person’s offence.
- Creating meaningful support networks
  - Promoting social inclusion and a sense of belonging by offering opportunities within a mixed-community whereby people from all different backgrounds and circumstances attend a service.
  - Having lived experience threaded throughout the service and within the staff team encourages a sense of community and belonging and promotes the value of equality.

**Supporting a Change of Identity Away From Offending:**
- Opportunities to learn new skills
  - Providing people with opportunities to learn new skills, supports them to realise their own potential.
  - Understanding that there is not a ‘one size fits all’ approach, therefore offering a variety of learning opportunities that provide life skills and work-readiness skills to support with growth and personal development.
- Specialised support and understanding of barriers faced by people with a sexual offending history
  - Having the knowledge and expertise about the complexities of this area of offending to achieve a non-judgemental approach towards people with sexual convictions.

**Allowing for the Space and Time to Realise Own Potential:**
- Open-ended support with no time constraints
  - The participants in this research had all been attending the programme for at least 3 years. By allowing an individual to set their own goals and within a timeframe of their choosing gives people the opportunity to truly develop and continually progress meaningfully through life skills and work-readiness.
- Personalised goal-focussed support
  - Working with an individual to identify their goals for the future can allow them to feel in control and make meaningful decisions about their own progression.
  - Providing support about how to navigate and overcome barriers they may face, where possible, due to the nature of their offending background.
  - Providing a positive structure and routine, which considers an individual’s wellbeing to allow them to achieve personalised goals in a realistic timeframe of their choosing.
  - Providing a person with the tools to navigate their own future and overcome future challenges in a healthy and meaningful way.

Incorporating all these recommendations may not be practical for some services but having an understanding of how to support and work with people with sexual convictions within services may help to support their recovery and rehabilitation.

## Data Availability

We are not able to make the data publicly available from this research project. Due to the nature of the participants’ offending, it was agreed that data would not be publicly shared in order to further protect the identity and anonymity of the participants and the community programme.

## Social Media Accounts

@ROgden_research
